# Transcriptional Response to Acute Thermal Exposure in Juvenile Chinook Salmon Determined by RNAseq

**DOI:** 10.1534/g3.115.017699

**Published:** 2015-04-24

**Authors:** Katharine M. H. Tomalty, Mariah H. Meek, Molly R. Stephens, Gonzalo Rincón, Nann A. Fangue, Bernie P. May, Melinda R. Baerwald

**Affiliations:** * Department of Animal Science, University of California, Davis, California 95616; † Department of Wildlife, Fish, and Conservation Biology, University of California, Davis, California 95616

**Keywords:** gene discovery, *Oncorhynchus tshawytscha*, Illumina, thermal tolerance

## Abstract

Thermal exposure is a serious and growing challenge facing fish species worldwide. Chinook salmon (*Oncorhynchus tshawytscha*) living in the southern portion of their native range are particularly likely to encounter warmer water due to a confluence of factors. River alterations have increased the likelihood that juveniles will be exposed to warm water temperatures during their freshwater life stage, which can negatively impact survival, growth, and development and pose a threat to dwindling salmon populations. To better understand how acute thermal exposure affects the biology of salmon, we performed a transcriptional analysis of gill tissue from Chinook salmon juveniles reared at 12° and exposed acutely to water temperatures ranging from ideal to potentially lethal (12° to 25°). Reverse-transcribed RNA libraries were sequenced on the Illumina HiSeq2000 platform and a *de novo* reference transcriptome was created. Differentially expressed transcripts were annotated using Blast2GO and relevant gene clusters were identified. In addition to a high degree of downregulation of a wide range of genes, we found upregulation of genes involved in protein folding/rescue, protein degradation, cell death, oxidative stress, metabolism, inflammation/immunity, transcription/translation, ion transport, cell cycle/growth, cell signaling, cellular trafficking, and structure/cytoskeleton. These results demonstrate the complex multi-modal cellular response to thermal stress in juvenile salmon.

Chinook salmon (*Oncorhynchus tshawytscha*) are native to the west coast of North America, ranging from Alaska to central California. A combination of overexploitation, habitat loss, and water diversions has contributed to significant declines in Chinook salmon populations in California over the past century (Yoshiyama *et al.* 2001). Thermal stress is a serious challenge facing the remaining Chinook salmon in California. Dams have played a substantial role in eliminating access to cooler historical habitats and have fundamentally altered the hydrology of most California rivers (Lindley *et al.* 2007; Poff *et al.* 2007). The majority of historic salmon habitats is now above impassible dams, restricting populations to lower elevations with higher ambient temperatures. The combination of increased climate variability (Lindley *et al.* 2007), restricted habitat, drought, and high water demands for human and agricultural use translates to an increased likelihood of thermal exposure for Chinook salmon (Brown and Bauer 2010).

Fall-run Chinook salmon juveniles inhabit freshwater for 1 to 7 months and are vulnerable to water temperature fluctuations during this period and during their migration to the ocean (Moyle 2002). Exposure to increased temperatures can cause behavioral and physiological changes in salmonids, including increased metabolic rates, increased oxygen consumption, erratic swimming behavior, and elevated heat shock protein levels (Barton and Schreck 1987; Mesa *et al.* 2002; Goniea *et al.* 2006; Bellgraph *et al.* 2010). These physiological and behavioral changes may place increased energy demands on fish and increase predator visibility, both of which can negatively impact juvenile survival. Temperature also affects smoltification, a set of physiological changes that prepare juvenile anadromous salmonids for the transition to salt water (Marine and Cech 2004). Optimal water temperatures for smoltification of California Chinook salmon are between 10° and 17.5°, and individuals that undergo smoltification at higher water temperatures experience reduced survival rates in salt water (Myrick and Cech 2000).

Transcriptional analysis is an important means for investigating the physiological response to environmental changes of nonmodel organisms, for which few genomic tools have been developed (Gracey 2007; Logan and Somero 2011; Garcia *et al.* 2012). Information from such studies can be particularly valuable in the conservation of threatened and endangered species, as in the case of the delta smelt, *Hypomesus transpacificus* (Connon *et al.* 2011a, b). Microarrays have been the predominant means of studying gene expression changes in salmonids following thermal stress (Arctic charr, *Salvelinus alpinus*) (Quinn *et al.* 2011), sockeye salmon, *Oncorhynchus nerka* (Jeffries *et al.* 2012; Jeffries *et al.* 2014), pink salmon, *Oncorhynchus gorbuscha* (Jeffries *et al.* 2014), rainbow trout, *Oncorhynchus mykiss* (Rebl *et al.* 2013), brown trout, *Salmo trutta* (Meier *et al.* 2014); to our knowledge, no other studies have examined the transcriptome-wide response of juvenile Chinook salmon to elevated temperatures. Here, we take an RNAseq approach to investigate gene expression changes associated with increased temperatures in juvenile Chinook salmon. This method offers a variety of improvements over microarrays for quantifying transcription. RNAseq allows for the examination of a large number of genes without necessitating prior knowledge of the gene sequences and it increases the accuracy of detection over a wide range of expression levels (Shendure 2008; Wang *et al.* 2009). Gill tissue was chosen for this experiment due to the complex physiological role of this organ and its rapid response to environmental stressors (Evans *et al.* 2005; Chou *et al.* 2008). The feasibility of nonlethal sampling of gill tissue also makes this a useful organ for future field studies (McCormick 1993; Schrock *et al.* 1994). By examining the suite of genes that are differentially expressed following thermal stress, we can identify groups of genes and cellular processes that may be important for responding to thermal stress in this species. This information may help us to identify thermal stress in wild fish through the use of gene expression assays, as well as provide candidate genes for the investigation of adaptation to thermal stress in future studies (see examples in Larsen *et al.* 2010; Wellband and Heath 2013).

## Materials and Methods

**Figure 1:**
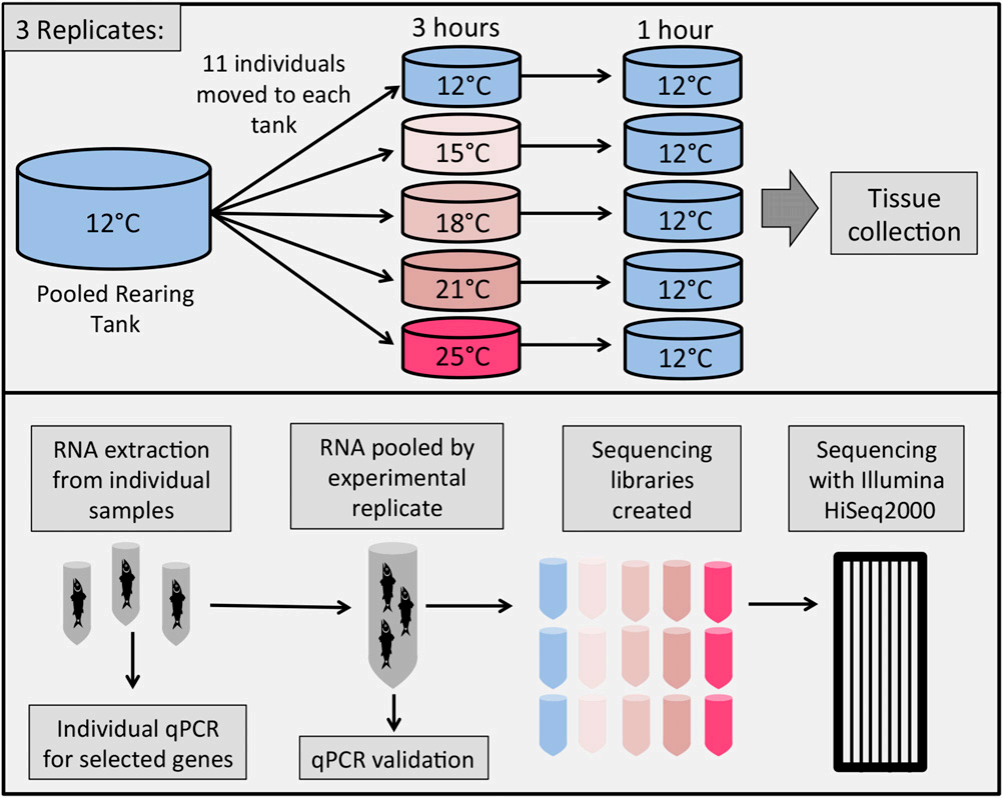
Experimental design.

Chinook salmon eggs obtained from Merced River Hatchery in November 2010 were reared at the University of California, Davis, in partially re-circulated aerated well water chilled to 12° (±1°) and fed commercial salmon feed (soft-moist formulation, 2–10% body weight; Rangen Inc.) until the time of the experiment, approximately 5 months posthatch. Fish were raised in a single 160-liter circular flow-through tank for 1 month preceding the experiment (see Figure 1 for experimental design), supplied with aerated well water and with a 12-hr light–12-hr dark photoperiod. The night prior to the experiment 55 fish were randomly assigned to one of five treatment groups (11 fish per treatment) and were allowed to acclimate at 12° in the experimental chambers overnight. Experimental chambers consisted of 5-gallon buckets with large mesh windows in the sides to allow water to freely enter and exit the chamber when submerged. Each chamber included an air stone to ensure well-aerated treatment water as well as to prevent thermal stratification. Water in the larger tanks was also well-aerated to maintain oxygen saturation. Treatments consisted of a 3-hr exposure to 15°, 18°, 21°, or 25° (±0.5°), followed by a 1-hr of recovery period at 12° (±0.5°). These temperatures range from optimal to the upper thermal limit for Chinook salmon determined by critical thermal methodology (Myrick and Cech 1998). Three hours was chosen as an ecologically relevant time exposure, because it approximates potential exposure times to warm water during juvenile outmigration and passage through warmer river reaches (Michel *et al.* 2013). The experimental chambers were moved to tanks held at a constant temperature using submersible titanium heating elements (Finnex 300W), facilitating very rapid change in the temperature experienced by the fish. Controls were handled identically to the other four treatment groups but remained at 12° (±0.5°). Following recovery, fish were killed with buffered tricaine methanesulfonate (500 mg/L tricaine and 420 mg/L NaCO_3_), weighed and measured, and gill tissue was immediately collected [there was no significant difference in weight (*P* = 0.53) or length (*P* = 0.79) of fish between groups]. Samples were preserved in RNAlater solution (Life Technologies) and stored at −80° prior to RNA extraction. Replicates of this temperature experiment were performed on 3 consecutive days at 9:00 am, yielding three replicates of 11 individuals at each temperature. Treatment of all animals was in accordance with University of California, Davis, animal care and use protocol #17875. RNA was isolated from gill tissue of 165 individuals using the TissueLyser II bead mill (Qiagen) for tissue homogenization and TRIzol Reagent Solution (Applied Biosystems) according to the manufacturer’s protocol. RNA was quantified and quality checked using the 2100 Bioanalyzer (Agilent) and RNA 6000 Nano Kit (Agilent). All RNA had a RIN (RNA Integrity Number) of 9.5 or higher. RNA from the 11 individuals in each experimental replicate was proportionally pooled and used to generate sequencing libraries using the Illumina TruSeq RNA Sample Preparation Kit and associated protocol (TruSeq RNA Sample Preparation Guide, part #15008136, November 2010 release). The 15 libraries were barcoded and processed, three libraries to a lane, with 100-bp paired-end sequencing on the Illumina HiSeq2000 platform at the University of California, Berkeley Vincent J. Coates Genomic Sequencing Laboratory. Raw data can be found at BioProject, record GSE59756.

The resulting sequences were quality-filtered (Phred quality score cutoff = 20), adapter sequences were removed from the beginning of each read, and 20 bp were trimmed from the end of each read to remove lower-quality bases (Cutadapt version 1.1). Trimmed reads were pooled and duplicates were removed before performing the *de novo* reference transcriptome assembly. Assembly was performed in CLC Genome Workbench (version 4.9 beta) using default settings for *de novo* assembly and a minimum contig length of 100 bp. Differentially expressed (DE) transcript identification was performed using CLC Genome Workbench (version 5.0). The sequences from each treatment group were then aligned back to the reference transcriptome and DE transcripts compared with the 12° control group were identified using Baggerly’s test with an FDR of 0.10 (Baggerly *et al.* 2003). The threshold for significance was set at *P* < 0.01 (FDR-corrected) and greater than two-fold change in expression. The *de novo* contigs were annotated with sequence descriptions identified through BLAST (Altschul *et al.* 1990) searches of the NCBI nucleotide database and assigned with GO terms, enzyme codes, KEGG pathways, and InterPro matches using default parameters in Blast2GO (Conesa *et al.* 2005).

Direction and magnitude of expression of three genes (SERPINH1, FOS, and CXCL8) in the pooled samples were confirmed with qPCR. RNA was treated with Deoxyribonuclease I, Amplification Grade (Life Technologies), to remove possible genomic DNA contamination according to manufacturer’s instructions. DNase-treated RNA was converted to cDNA using the SuperScript VILO cDNA Synthesis Kit (Life Technologies) according to the manufacturer’s instructions. Quantitative PCR was then performed using BioRad Chromo4 real-time detector in an 8 μl reaction [1 μM forward primer, 1 μM reverse primer, 1X Quantitect SYBR Green (Qiagen), and 5 ng cDNA]. Cycling parameters were 95° (15 min), 94° (15 sec), 58° (30 sec), 72° (30 sec) (data acquisition) × 45 cycles, followed by a melt curve between 55° and 95° to ensure a single amplicon was present (Table 1). Standard curves were performed for each primer pair to ensure PCR efficiency was between 95% and 105%. Three replicates were performed for each sample and two no-template controls were included on each plate, and a no-RT control was included for each sample. Quantitative PCR was also performed for selected genes on individual RNA samples to assess expression variation within a pooled treatment group (Supporting Information, Figure S1). Quantitative PCR results were normalized to the housekeeping gene EF1A after ensuring its expression level remained stable across samples and experimental conditions. Analysis of qPCR data was performed using the delta delta Ct method, and results for all three technical replicates for a given sample were required to be within 10% of each other to be included.

**Table 1:** Primer sequences and GenBank accession numbers for gene expression validated by qPCR

Gene Group	Gene Name	Gene Symbol	GenBank Accession Number	Primers (5′-3′)
Protein folding	Heat shock protein 47	SERPINHI	AB196463.1	F-GTTCCCATGATGCATCGCAC
				R-CCTTGGTTTTGTCCACAGCG
Transcription	C-fos	FOS	AB111054.1	F-AATGACTTTGAGCCCCTGTG
				R-GTAGGGGAGCTGAGGGAATC
Inflammation/ immunity	Chemokine ligand 8	CXCL8	AY160982.1	F-AGAATGTCAGCCAGCCTTGT
				R-CTTGCTCAGAGTGGCAATGA
Housekeeping	Elongation factor 1A	EF1A	FJ890356.1	F-TCTCAGGCTGATTGCGCTGT
				R-GGGGGCTCAGTAGAGTCCAT

## Results and Discussion

Sequencing yielded 885,283,684 raw reads, of which 836,304,468 remained after quality trimming and filtering. The *de novo* transcriptome generated from the filtered reads consisted of 183,778 contigs and was used as the reference for identification of differential gene expression at the four experimental temperatures. Four contigs were upregulated at 15°, 104 at 18°, 697 at 21°, and 3976 at 25°. Five contigs were downregulated at 15°, 16 at 18°, 101 at 21°, and 6166 at 25°. Of the upregulated contigs, an average of 48.4% were identified through a BLAST search as known sequence in the NCBI database, 41.5% were assigned Gene Ontology (GO) terms, and 46.7% matched a known protein in the InterPro database (Table 2, Table 3). Quantitative reverse-transcription PCR of three genes verified the direction and relative magnitude of expression change obtained through RNAseq (Table 4). A full gene list of all upregulated and downregulated genes is given in Table S1. Slight variations between the fold-change found by RNAseq and RT-qPCR protocols are expected due to the fact that the RNAseq samples were pooled at the RNA stage, whereas the qPCR was performed on individual cDNA samples and the results were then pooled. Pooling samples at the RNA stage is advantageous in that treatment means can be more accurately estimated and costs can be minimized; however, it precludes an evaluation of interindividual variation (Konczal *et al.* 2013). Future studies designed to estimate interindividual variation will be complementary to our results presented here. Additionally, although paralogues are common in salmonid species due to their genome structure, identification of paralogues was outside the scope of this study. These data are presented with the caveat that a portion of the results will be showing expression for more than one gene paralogue.

**Table 2:** Summary statistics of Illumina sequencing results

	12°	15°	18°	21°	25°
Raw sequences	192,559,160	163,018,834	189,541,778	178,501,904	161,662,008
Sequences after QC filtering	182,390,970	154,294,234	179,245,463	167,554,816	152,818,985
DE contigs upregulated		4	104	697	3976
Upregulated contigs annotated by BLAST		1 (25%)	55 (53%)	354 (51%)	1902 (48%)
Upregulated contigs assigned GO terms		1 (25%)	54 (52%)	322 (46%)	1608 (40%)
Upregulated contigs assigned Enzyme Codes		0	5 (5%)	70 (10%)	487 (12%)
Upregulated contigs annotated by InterProScan		1 (25%)	50 (48%)	347 (50%)	1834 (46%)
Upregulated KEGG pathways		0	2	13	56
Upregulated KEGG Pathways with >2 enzymes		0	0	5	25
DE contigs downregulated		5	16	101	6166
Downregulated contigs annotated by BLAST		2 (40%)	5 (31%)	40 (40%)	3481 (56%)
Downregulated contigs assigned GO terms		2 (40%)	3 (12%)	31 (31%)	2972 (48%)
Downregulated contigs assigned Enzyme Codes		0	0	8 (8%)	626 (10%)
Downregulated contigs annotated by InterProScan		2 (40%)	9 (56%)	45 (45%)	—
Downregulated KEGG pathways		0	0	1	—
Downregulated KEGG pathways with >2 enzymes		0	0	0	—

The number of contigs that were upregulated and downregulated at each temperature and were identified through a BLAST search of the NCBI database, assigned GO terms, assigned Enzyme Codes, or annotated through InterProScan. The percent of filtered sequences meeting each criterion follows in parentheses. Number of KEGG pathways for which an enzyme was differentially expressed is listed. *For two-fold change with a *P* < 0.01 and FDR of 0.10.

**Table 3:** Gene Ontology terms identified by Blast2GO analysis for contigs significantly upregulated at 25° (FDR 0.1, *P* < 0.01, >2-fold change)

GO Category	GO ID	Name	GO ID	Name	# Contigs
Biological Process	GO:0071840	Cellular component organization or biogenesis	GO:0016043	Cellular component organization	124
			GO:0007005	Mitochondrion organization	18
			GO:0006996	Organelle organization	50
	GO:0009987	Cellular process	GO:0007154	Cell communication	11
			GO:0007049	Cell cycle	87
			GO:0008219	Cell death	129
			GO:0019725	Cellular homeostasis	34
			GO:0006464	Cellular protein modification process	175
			GO:0007010	Cytoskeleton organization	85
			GO:0007165	Signal transduction	337
			GO:0006412	Translation	27
	GO:0032502	Developmental process	GO:0009653	Anatomical structure morphogenesis	202
			GO:0030154	Cell differentiation	152
			GO:0009790	Embryo development	87
			GO:0007275	Multicellular organismal development	315
	GO:0040007	Growth	GO:0016049	Cell growth	21
	GO:0008152	Metabolic process	GO:0009058	Biosynthetic process	155
			GO:0005975	Carbohydrate metabolic process	27
			GO:0009056	Catabolic process	170
			GO:0006259	DNA metabolic process	43
			GO:0006091	Generation of precursor metabolites and energy	11
			GO:0006629	Lipid metabolic process	68
			GO:0006139	Nucleobase-containing compound metabolic process	127
			GO:0044238	Primary metabolic process	6
			GO:0019538	Protein metabolic process	216
			GO:0019748	Secondary metabolic process	6
	GO:0051704	Multi-organism process	GO:0044403	Symbiosis, encompassing mutualism through parasitism	20
			GO:0016032	Viral process	20
	GO:0050789	Regulation of biological process	GO:0040029	Regulation of gene expression, epigenetic	4
	GO:0000003	Reproduction			91
	GO:0050896	Response to stimulus	GO:0007610	Behavior	33
			GO:0009628	Response to abiotic stimulus	51
			GO:0009607	Response to biotic stimulus	83
			GO:0009719	Response to endogenous stimulus	46
			GO:0009605	Response to external stimulus	100
			GO:0006950	Response to stress	237
	GO:0044699	Single-organism process	GO:0008283	Cell proliferation	91
			GO:0008037	Cell recognition	26
			GO:0007267	Cell–cell signaling	18
	GO:0006810	Transport	GO:0006811	Ion transport	52
			GO:0015031	Protein transport	66
Molecular Function	GO:0016209	Antioxidant activity			7
	GO:0005488	Binding	GO:0003779	Actin binding	34
			GO:0005509	Calcium ion binding	51
			GO:0030246	Carbohydrate binding	11
			GO:0003682	Chromatin binding	22
			GO:0008092	Cytoskeletal protein binding	20
			GO:0003677	DNA binding	142
			GO:1901363	Heterocyclic compound binding	1
			GO:0008289	Lipid binding	33
			GO:0003676	Nucleic acid binding	48
			GO:0000166	Nucleotide binding	262
			GO:0097159	Organic cyclic compound binding	1
			GO:0005515	Protein binding	498
			GO:0005102	Receptor binding	83
			GO:0003723	RNA binding	42
			GO:0008135	Translation factor activity, nucleic acid binding	5
	GO:0003824	Catalytic activity	GO:0016787	Hydrolase activity	98
			GO:0016301	Kinase activity	15
			GO:0003774	Motor activity	2
			GO:0004518	Nuclease activity	6
			GO:0008233	Peptidase activity	46
			GO:0004721	Phosphoprotein phosphatase activity	26
			GO:0004672	Protein kinase activity	58
			GO:0016740	Transferase activity	89
	GO:0009055	Electron carrier activity			19
	GO:0030234	Enzyme regulator activity			66
	GO:0060089	Molecular transducer activity	GO:0004871	Signal transducer activity	13
	GO:0001071	Nucleic acid binding transcription factor activity	GO:0003700	Sequence-specific DNA binding transcription factor activity	95
	GO:0004872	Receptor activity			43
	GO:0005198	Structural molecule activity			10
	GO:0005215	Transporter activity	GO:0005216	Ion channel activity	6
Cellular Component	GO:0030313	Cell envelope			1
	GO:0071944	Cell periphery	GO:0030312	External encapsulating structure	1
			GO:0005886	Plasma membrane	134
	GO:0005737	Cytoplasm	GO:0016023	Cytoplasmic membrane-bounded vesicle	68
			GO:0005829	Cytosol	134
			GO:0005811	Lipid particle	9
	GO:0005576	Extracellular region	GO:0005615	Extracellular space	38
			GO:0005578	Proteinaceous extracellular matrix	22
	GO:0043226	Organelle	GO:0005694	Chromosome	37
			GO:0005929	Cilium	4
			GO:0005856	Cytoskeleton	82
			GO:0005783	Endoplasmic reticulum	115
			GO:0005768	Endosome	19
			GO:0072546	ER membrane protein complex	1
			GO:0005794	Golgi apparatus	48
			GO:0005764	Lysosome	6
			GO:0005815	Microtubule organizing center	32
			GO:0005739	Mitochondrion	78
			GO:0000228	Nuclear chromosome	20
			GO:0005635	Nuclear envelope	14
			GO:0005730	Nucleolus	86
			GO:0005654	Nucleoplasm	166
			GO:0005634	Nucleus	233
			GO:0005777	Peroxisome	3
			GO:0005840	Ribosome	8
			GO:0005773	Vacuole	5
	GO:0043234	Protein complex			290

The three GO categories, Biological Process, Molecular Function, and Cellular Component, are presented along with GO terms of children and number of contigs assigned each term.

**Table 4:** Fold change of selected genes as determined by RNAseq and quantitative reverse-transcription PCR

Gene	15**°**	18**°**	21**°**	25**°**
SERPINH1 (RNAseq)	<2	7	17	34
SERPINH1 (qPCR)	4.9	16.9	38.4	43.3
FOS (RNAseq)	<2	<2	3	24
FOS (qPCR)	1.1	1.1	3.4	43.7
CXCL8 (RNAseq)	<2	<2	<2	13
CXCL8 (qPCR)	1.1	0.9	1.1	18.3

Under thermal stress, especially at higher temperatures, normal cellular functions are suspended as the organism attempts to cope with the current stress (Parsell and Lindquist 1993; Kültz 2005; Logan and Somero 2011). Twenty-six KEGG pathways were identified for which at least two enzymes were upregulated (Table 5). Based on GO terms and protein functional information obtained from the UniProt database, 12 manually curated functional gene groups were identified among the results: (1) protein folding/rescue; (2) protein degradation; (3) cell death; (4) oxidative stress; (5) metabolism; (6) inflammation/immunity; (7) transcription/translation; (8) ion transport; (9) cell cycle/growth; (10) cell signaling; (11) cellular trafficking; and (12) structure/cytoskeleton. These functional categories represent a picture of biologically relevant changes that take place in response to acute thermal exposure in Chinook salmon. We provide interpretation of the gene function based on GO terms, the UniProt database, and published literature, although there may be additional functional roles of the identified genes that are not outlined here. We focused our discussion on gene functions to those that have been identified in fish or shown to be related to thermal stress. Likewise, we emphasize the roles of upregulated transcripts, although relevant downregulated genes also exist (Table S1).

**Table 5:** List of KEGG pathways for which at least two enzymes are differentially expressed at 25°

KEGG Pathways Upregulated at 25°		KEGG Pathways Downregulated at 25°	
Pathway	# Enzymes	Pathway	# Enzymes
Purine metabolism	7	Purine metabolism	22
One carbon pool by folate	6	Phosphatidylinositol signaling system	11
Arginine and proline metabolism	5	Glycerophospholipid metabolism	10
Amino sugar and nucleotide sugar metabolism	5	Amino sugar and nucleotide sugar metabolism	9
Glycine, serine, and threonine metabolism	4	Inositol phosphate metabolism	9
Pyrimidine metabolism	4	Pyrimidine metabolism	8
Arachadonic acid metabolism	4	Pyruvate metabolism	8
Cysteine and methionine metabolism	4	Citrate cycle	7
Mucin type O-glycan biosynthesis	4	Pentose and glucuronate interconversions	7
Fructose and mannose metabolism	4	Fructose and mannose metabolism	7
Lysine degradation	3	Glycine, serine, and threonine metabolism	7
Aminoacyl-tRNA biosynthesis	3	Glutathione metabolism	7
Sphingolipid metabolism	3	Glycerolipid metabolism	7
Terpenoid backbone biosynthesis	3	Carbone fixation pathways in prokaryotes	7
Other types of O-glycan biosynthesis	3	Alanine, aspartate, and glutamate metabolism	6
Starch and sucrose metabolism	3	Tryptophan metabolism	6
Carbon fixation pathways in prokaryotes	3	Arachadonic acid metabolism	6
T-cell receptor signaling pathway	2	Porphyria and chlorophyll metabolism	6
Porphyrin and chlorophyll metabolism	2	Glycolysis/gluconeogenesis	5
Steroid biosynthesis	2	Pentose phosphate pathway	5
Glycosphingolipid biosynthesis—lacto and neglector series	2	Arginine and proline metabolism	5
Drug metabolism—other enzymes	2	Glycosylate and dicarboxylate metabolism	5
Alanine, aspartate and glutamate metabolism	2	Butanoate metabolism	5
Folate biosynthesis	2	Aminoacyl-tRNA biosynthesis	5
Linoleic acid metabolism	2	Galactose metabolism	4
		Fatty acid degradation	4
		Valine, leucine, and isoleucine degradation	4
		Lysine degradation	4
		Starch and sucrose metabolism	4
		Glycosaminoglycan degradation	4
		Ether lipid metabolism	4
		Sphingolipid metabolism	4
		Methane metabolism	4
		Drug metabolism	4
		Ascorbate and alderate metabolism	3
		Synthesis and degradation of ketone bodies	3
		Steroid hormone biosynthesis	3
		Cysteine and methionine metabolism	3
		Beta-alanine metabolism	3
		Various types of N-glycan biosynthesis	3
		Alpha-linolenic acid metabolism	3
		Glycosphingolipid biosynthesis-globoseries	3
		Propanoate metabolism	3
		One carbon pool by foliate	3
		Retinol metabolism in animals	3
		Metabolism by xenobiotics by cytochrome P450	3
		Drug metabolism—cytochrome P450	3
		Geraniol degradation	2
		Histidine metabolism	2
		Tyrosine metabolism	2
		Phenylalanine metabolism	2
		Cyanoamino acid metabolism	2
		N-glycan biosynthesis	2
		Peptidoglycan biosynthesis	2
		Linoleic acid metabolism	2
		Chloroalkane and chloroalkene degradation	2
		Aminobenzoate degradation	2
		Carbone fixation in photosynthetic organisms	2
		Nicotinate and nicotinamide metabolism	2
		Nitrogen metabolism	2
		Phenylpropanoid biosynthesis	2
		mTOR signaling pathway	2
		T-cell receptor signaling pathway	2

Several KEGG pathways are represented in both the upregulated and downregulated groups. For these common pathways, different enzymes are upregulated or downregulated.

### Management of denatured proteins

Protein denaturation and agglutination are well-studied consequences of thermal stress and can disrupt normal cell function (reviewed in Dewey 1989; Nguyen *et al.* 1989; Nover 1991; Lepock 2004; Gsponer and Babu 2012). There are two classic ways in which the cell responds to denatured proteins, either through rescue and stabilization by chaperones, such as the heat shock family of proteins (reviewed in Lindquist 1986; Kampinga 2006), or by degradation of damaged proteins through the ubiquitin/proteosome pathway (reviewed in Glickman and Ciechanover 2002). In juvenile Chinook salmon, the expression of protein folding/rescue genes was upregulated starting at 18° (two-fold to six-fold) and was highest in the 25° group (two-fold to 2150-fold), whereas protein degradation-associated genes were only expressed in the 21° (two-fold to four-fold) and 25° (two-fold to 24-fold) groups. This is consistent with the expected response whereby at lower temperatures proteins are more easily salvaged through chaperone activity, and only at higher temperatures do unsalvageable proteins start to be degraded as the impact of thermal stress becomes more critical (Logan and Somero 2011).

**Figure 2:**
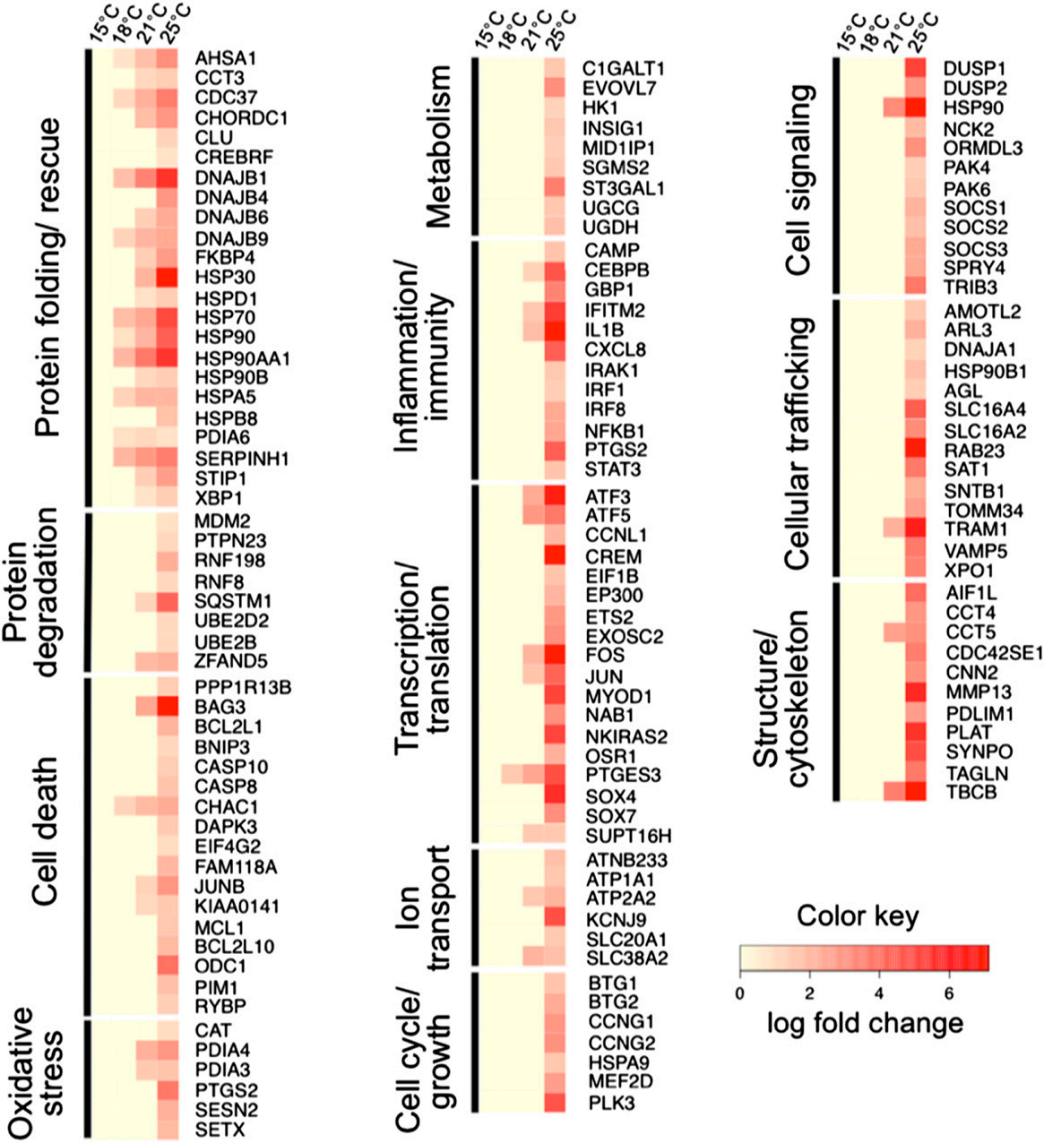
Heat map showing the log fold change of selected upregulated genes at each experimental temperature. The genes are clustered into functional gene groups based on Gene Ontology and UniProt information. The genes fall into one of 12 functional groups: (1) protein folding/rescue; (2) protein degradation; (3) cell death; (4) oxidative stress; (5) metabolism; (6) inflammation/immunity; (7) transcription/translation; (8) ion transport; (9) cell cycle/growth; (10) cell signaling; (11) cellular trafficking; and (12) structure/cytoskeleton.

Protein chaperones play a vital role under both normal and stress conditions by refolding or stabilizing proteins in their correct conformation (reviewed in Gething and Sambrook 1992; Hartl 1996) and are highly conserved among eukaryotes (reviewed in Feder and Hofmann 1999). Members of the heat shock protein family are expressed constitutively in various tissues (Fader *et al.* 1994; Boone and Vijayan 2002), whereas others are expressed as part of the general response to a variety of stressful stimuli including (but not limited to) temperature, chemical contaminants, hypoxia, pursuit by predators, and social hierarchy dynamics (reviewed Iwama *et al.* 1999; Basu *et al.* 2002; Iwama *et al.* 2004). Chaperone-coding genes were some of the most robustly expressed genes in this experiment and were expressed at temperatures as low as 18° (Figure 2). Heat shock proteins are often divided into general categories by molecular weight. Three of the most common are the HSP90 family, HSP70 family, and small HSPs. Expression of *HSP90* gene isoforms was upregulated at 18°, 21°, and 25°, as were many known co-chaperones of HSP90, including *CDC37*, *AHSA1*, *FKBP4*, *CHORDC1*, *HSP5A*, and *STIP1*.

HSP90s are involved in both normal cellular function and the cellular stress response and act as protein-stabilizing chaperones (*e.g.*, Wiech *et al.* 1992; Jakob *et al.* 1995; reviewed in Saibil 2013). The CDC37 protein physically interacts with the HSP90 protein and modifies its function by the recruitment of other co-chaperones (Roe *et al.* 2004; Röhl *et al.* 2013). The product of the *AHSA1* gene (also known as *AHA1*) interacts with the HSP90 protein and can increase its ATPase activity by up to five-fold (Lotz *et al.* 2003; Röhl *et al.* 2013). The CHORDC1 protein interacts physically with HSP90 *in vitro* (Gano and Simon 2010). HSP5A (also known as GRP78) forms multimeric complexes with HSP90 and PDIA6, as well as plays a key role in the unfolded protein response (UPR) (Tiffany-Castiglioni and Qian 2012). HSP90 has also been found to interact with co-chaperone PDIA6 in the skeletal muscle of Atlantic salmon (de La Serrana and Johnston 2013). Interestingly, CDC37, HSP90, FKBP4, and AHSA1 have been shown to play an important role in RNAi-mediated gene silencing in mammals (Pare *et al.* 2013). Oxidative stress can induce RNAi silencing and subsequently reduced thermal tolerance in *C. elegans* (Spiró *et al.* 2012). Examining the role of RNAi in the stress response of fish is a potentially interesting line of future inquiry. The upregulation of *CDC37*, *HSP90*, *FKBP4*, and *AHSA1* is likely functioning to stabilize denatured proteins in the experimental fish.

Expression of *HSP70* and associated co-chaperones in the DNAJ family (also known as *HSP40*) were upregulated at 18°, 21°, and 25°. HSP70 is an important chaperone protein that stabilizes and refolds proteins in their correct conformation under stress conditions (reviewed in Silver and Noble 2012). Members of the DNAJ family of proteins regulate the function of HSP70 by stabilizing the interaction between HSP70 and the target proteins (Mayer and Bukau 2005; Qui *et al.* 2006).

Expression of the small HSPs, *HSP30* and *HSPB8* (also known as *HSP22*), along with the functionally similar *CLU*, was upregulated at 25° (Carver *et al.* 2003). *HSP30* is notable because it was upregulated by 1250.1-fold in the highest temperature group. *SERPINH1* gene (also known as *HSP47*) was strongly upregulated (34.3-fold) and encodes a protein that is essential for normal growth and development, but that also plays an important role in collagen stabilization under stress conditions (reviewed in Nagata 1996). X-box binding protein (coded by *XPB1* gene), upregulated in the 21° and 25° groups, is a transcription factor that regulates expression of chaperone-coding genes during the UPR in the endoplasmic reticulum (Lee *et al.* 2003). HSP60, another upregulated gene product, functions as a protein chaperone but also may play a role in apoptosis by forming a pro-apoptotic complex with procaspase 3 (Samali *et al.* 1999). The large number of strongly upregulated chaperone-coding genes supports the conclusion that protein folding and stabilization are important heat stress coping mechanisms in juvenile Chinook salmon.

The ubiquitin/proteasome pathway is the other major destination for unfolded or damaged proteins that are not salvaged through chaperone stabilization or refolding. Proteins are marked for removal by the covalent attachment of multiple ubiquitin molecules and are subsequently degraded by the 26S proteasome or the lysosome (Ciechanover 1994; Ciechanover 1998). Multiple genes involved in the attachment of ubiquitin to target proteins were upregulated in juvenile Chinook salmon at 25°, including the ubiquitin ligases RNF19B and RNF8 and the ubiquitin conjugating enzyme UBE2B, which attach ubiquitin molecules to target proteins. RNF19B and the ubiquitin carrying protein type E2 are both associated with inflammatory and immune activation by cytotoxic t-cells or the NF-κB pathway, respectively (Gonen *et al.* 1999; Kozlowski *et al.* 1999). Genes (*SQSMT1*, *ZFAND5*, *PTPN23*) associated with the formation of polyubiquitinated bodies and the endosomal sorting machinery that processes targeted proteins for lysosomal degradation were also upregulated in individuals exposed to the 21° and 25° treatments (Pankiv *et al.* 2007; Doyotte *et al.* 2008; Garner *et al.* 2011; Shields and Piper 2011; Ali *et al.* 2013). The ubiquitin/proteosome pathway is likely strongly active in the experimental fish, especially in the higher temperature groups, indicating that the need for disposal of denatured proteins begins very early in the thermal stress response in these fish.

### Cell death

Chinook salmon from the 18° treatment show a slight (two-fold to three-fold) increase in apoptosis-related gene expression, which increases up to seven-fold in the 21° treatment group. The 25° treatment group demonstrates a large increase in expression and number of upregulated apoptosis-related genes, ranging from two-fold to 220-fold change. Twelve out of 33 individuals in the 25° group became unresponsive and/or died during the 3-hr exposure. These individuals were monitored closely and removed for sampling promptly after cessation of opercular beats to avoid degradation of RNA in the sampled tissues.

At least 18 pro-apoptotic genes were upregulated by Chinook salmon following thermal exposure. The upregulation of *CASP8*, *CASP10*, *RYBP*, and *KIAA0141* in the 25° group indicates that the intrinsic apoptotic pathway is active. Caspases 8 and 10 both participate in apoptotic cascades as well as increase NF-κb-mediated inflammation (Wang *et al.* 2001; Takahashi *et al.* 2005; Sakata *et al.* 2007). KIAA0141, death-associated protein effector DELE, is a pro-apoptotic protein that functions in the same pathway as caspases 8 and 10, and is functionally enhanced by inflammatory proteins such as TNFα (Harada *et al.* 2010). *RYBP* codes for death effector domain-associated factor, which binds with the protein hippy to increase the apoptotic effect of caspase 8 (Stanton *et al.* 2007). The apoptotic and inflammatory responses may be tightly intertwined during the response to stress observed in juvenile Chinook salmon. *PPP1R13B*, *JUNB*, *ODC1* code for apoptosis-inducing proteins and were upregulated at 21° and 25° (Jacobs-Helber *et al.* 1998; Samuels-Lev *et al.* 2001; Bergamaschi *et al.* 2004). Interestingly, increases in ODC1 have been observed in moribund temperature-stressed adult salmonids (Pignatti *et al.* 2004), and it has functional roles beyond apoptosis, including involvement in cell proliferation.

There is some overlap between the UPR and induction of apoptosis (Didelot *et al.* 2006). *CHAC1* (upregulated at 18°, 21°, and 25°) is a pro-apoptotic gene that is upregulated in response to the UPR (Mungrue *et al.* 2008). BAG3 (upregulated at 21° and 25°) is a regulator of many biological processes, among them apoptosis, and is a member of the BCL2 co-chaperone family that interacts directly with HSP70 (Rosati *et al.* 2011). Due to the multiple roles of these genes, it is uncertain which ultimate function they would serve in temperature-stressed fish in this experiment.

The processes of apoptosis and autophagy are tightly linked. Apoptosis results in cell death (type I), whereas autophagy can aid a cell in preventing apoptosis or can result in autophagic death (type II). Autophagy and apoptosis are mutually inhibitory to some extent, and both processes may be competing in a stressed cell (Mauiri *et al.* 2007). Given the mutual inhibition of autophagy and apoptosis, it is not surprising that we found upregulation of several genes that code for anti-apoptotic proteins, in addition to the pro-apoptotic genes discussed above. Genes in the BCL2 family regulate apoptosis and can be either pro- or anti-apoptotic. *BCL2L1*, *BNIP3*, *MCL1*, and *BCL2L10* are all generally anti-apoptotic members of this family and were upregulated in the 25° treatment group (Lee *et al.* 0.1999; Desagher and Martinou 2000; Vande Velde *et al.* 2000; Sevilla *et al.* 2001; Craig 2002). Upregulated PIM1, another regulator of apoptosis, is usually anti-apoptotic, but it has also been shown to be pro-apoptotic in other circumstances (Lilly *et al.* 1999; Mochizuki *et al.* 1997; Aho *et al.* 2004; Gu *et al.* 2009).

### Oxidative stress

At least six genes involved in the oxidative stress response were upregulated after 25° exposure. This is most likely due to a general upregulation of protective compounds during the cellular stress response, because water in the experimental chambers was well-aerated and the fish were not exposed to hypoxic conditions. The upregulated genes *CAT* and *SESN2* encode proteins that protect cells from oxidative damage due to hydrogen peroxide and other oxidative compounds (Takeuchi *et al.* 1995; Budanov *et al.* 2004, 2011). The gene *PDIA3* (two- to three-fold) was upregulated in Chinook salmon in the 25° group has been shown in previous studies to increase in Atlantic salmon (*S. salar*) smolts held in hypoxic conditions (Huang *et al.* 2009. One isoform of the *COX2* gene, *PDGS2A*, was upregulated in the 25° group (eight-fold). Interestingly, zebrafish have two inducible isoforms of the *COX2* gene that share some functional overlap in response to oxidative stress and inflammatory conditions (Ishikawa *et al.* 2007) and may indicate a link between the response to oxidative stress and inflammation (Uchida 2008).

### Inflammation/immunity

We saw several indications of an inflammatory response as well as an increase in innate immune activity, particularly in the 25° group of fish (two-fold to 67-fold). Acute thermal stress has been shown to induce the classic vertebrate inflammatory response in other fish species, such as the Antarctic plunderfish, *Harpagifer antarcticus* (Thorne *et al.* 2010), and innate immune activity increases following acute stress events in many fish species (Tort 2011). Expression of a number of classic inflammatory mediators was upregulated, such as the cytokines chemokine (C-C-C motif) ligand 8 (*CXCL8*) and interleukin 1 beta (*IL1B*), and the cellular transducer of interleukin signaling gene *STAT3* (Van Damme *et al.* 1985; Mukaida 2000; Levy and Lee 2002). The *COX2* gene was also upregulated, which codes for a member of the biosynthetic pathway that produces inflammatory thromboxanes and prostaglandins (Rouzer and Marnett 2009). There is a large degree of overlap between inflammatory and innate immune responses. Many of the molecular players are functionally intertwined and the full extent of the relationship between the two responses is not yet fully understood (Medzhitov 2008; Muralidharan and Mandrekar 2013). The upregulated gene *CEBPB*, coding for CCAAT/enhancer-binding protein beta, increases expression of both immune and inflammatory proteins such as NF-κB and cycloxygenase 2 (Mercurio and Manning 1999; Zhu *et al.* 2002; Park *et al.* 2010). *IRAK1*, also upregulated, plays a central role in mediating the immune response and is activated by the inflammatory cytokine IL1 (Gottipatti *et al.* 2008). Several genes coding for antimicrobial proteins were upregulated, including *CAMP* and the antiviral compound-coding genes *GPB1* and *IFITM2* (Anderson *et al.* 1999; Wang *et al.* 2008; Siegrist *et al.* 2011).

### Ion transport

Several types of ion-linked transporters were upregulated in the 21° and 25° groups, which may be of interest for three reasons. First, increases in cellular ion concentrations, particularly of calcium, are important for the UPR. Second, a number of transporters are upregulated by immune/inflammatory genes and presumably play a role in those cellular responses. Third, gills are an important osmoregulatory organ for anadromous salmonids that adjust throughout different life stages to either fresh or saltwater environments (Evans 2002). Although salinity was not a factor in this experiment, the fish were nearing the age at which smoltification would typically begin and were therefore in a dynamic period of ion pump expression. Two genes coding for subunits of the sodium/potassium-transporting ATPase were upregulated, *ATNB233* and *ATP1A1*, along with a G-protein-activated inward potassium channel, *KCNJ9*. The upregulated gene *SLC20A1* codes for a sodium-dependent phosphate transporter that is correlated with cytokine and IL-alpha increases (Sabatino *et al.* 1997). *SLC20A1* is expressed in most cells and may play a separate role as a negative regulator of TNF-mediated apoptosis (Salaün *et al.* 2010). *ATP2A2* (also known as *SERCA2*) expression was upregulated at both 21° and 25°. The sarcoplasmic reticulum calcium pump encoded by this gene is important for maintaining calcium homeostasis in the endoplasmic reticulum and has been linked to the UPR and concurrent increased need for calcium ions (Okada *et al.* 2002; Schröder 2008). Upregulated gene *SLC38A2* (also known as *SNAT2*) codes for a sodium-coupled neutral amino acid transporter that has been linked with recovery from hypertonic stress in human cells (Bevilacqua *et al.* 2005).

### Metabolic changes

Cells under thermal stress presumably halt the synthesis of nonessential proteins and molecules and put cellular resources toward the synthesis of products that play a role in coping with heat stress. Glycolipid and glycoprotein biosynthetic pathways were found to be upregulated by thermal stress, particularly in the 25° group. Eight KEGG pathways involved in the synthesis of glycolipids or glycoproteins contained at least two upregulated genes (Table 5). Glycolipids play a number of biological roles in the cell, including physical defense, cell–cell signaling, membrane–protein interactions, and immune function (Varki *et al.* 1999). Temperature has been shown to change the composition of glycosphingolipids in the plasma membrane of some eukaryotes (Aaronson and Martin 1983). Ceramide in particular is a glycosphingolipid that plays a prominent role in the cellular stress response, affecting cell–cell signaling and apoptosis (Hannun 1996; Hussain *et al.* 2012). *UGCG* and *SGMS2* were upregulated in the 25° group and participate in ceramide biosythesis (Ichikawa *et al.* 1996; Vermeil *et al.* 1996). Upregulated *CIGALT1B* codes for a chaperone required for some o-glycan biosynthesis. O-glycans play many roles in the cell, including cell signaling (Ju and Cummings 2002).

Another role of glycolipids that is of note is the production of mucus by epithelial cells. Mucus can act as physical defense for epithelial cells by forming a gelatinous barrier, and may function to protect the gill epithelium from physical damage during exposure to thermal stress (Shephard 1994; Loganathan *et al.* 2013).

### Transcription/translation

Unsurprisingly, expression of many regulators of transcription and translation was altered. Several transcription factors relating to stress, UPR, apoptosis, and inflammation were upregulated in the 21° and 25° groups. ATF5, for instance, is a transcription factor that is implicated in production of small HSPs (Wang *et al.* 2007). *ATF3* is similarly known to be a stress response gene that is induced by physiological stress (Chen *et al.* 1996). Upregulated *EP300* stabilizes transcription factor HSF-1, which in turn regulates expression of heat shock proteins and chaperones (Raychaudhuri *et al.* 2014). FOS can function with the JUN/AP1 complex to regulate transcription (Zhang *et al.* 1998).

Several transcription factors are stimulated by DNA damage or oxidative stress to increase transcription of various stress response genes and were upregulated. *ETS2* expression is activated by oxidative stress, whereas the SOX4 and SUPT16H proteins sense DNA damage and regulate the p53 and FACT complexes, respectively (Lee *et al.* 2009; Pan *et al.* 2009; Dinant *et al.* 2013). NKIRAS2 is a transcription factor that regulates the activity of NF-κB through regulation of its inhibitor, IκB (Fenwick *et al.* 2000). PTGES3 (also known as p23) is a central regulator of transcriptional complexes involved in stress response (Freeman and Yamamoto 2002). The changes in transcription factor expression appear to be related in large part to apoptotic regulation and chaperone expression and function.

### Cell cycle/cell growth

Expressions of the cell cycle and growth-related genes *BTG1*, *BTG2*, *CCNG1*, *CCNG2 PLK3*, and *HSPA9* were upregulated at 25° (two-fold to nine-fold), demonstrating a strong increase in antiproliferative genes. BTG1 and BTG2 both decrease cell proliferation, and BTG2 is part of the p53 DNA damage response pathway (Rouault *et al.* 1992; Rouault *et al.* 1996). CCNG1 and CCNG2 (cyclin G1 and G2) are induced by DNA damage and halt the cell cycle at G1 and G2 phases, respectively. CCNG1 is also part of the p53 pathway, whereas CCNG2 is independent of this response (Bates *et al.* 1996). The stress-induced gene *PLK3* encodes a kinase that arrests the cell cycle and is known to be upregulated by peroxide exposure (Bahassi *et al.* 2002). Mortalin (encoded by *HSPA9*) is a heat shock protein that serves many functions under stress conditions, including cell cycle arrest (Kaul *et al.* 2002). Although there was no significant change in genes controlling cell proliferation at the lower temperatures, the increase in antiproliferative genes observed at 25° indicates and halting of normal cell growth under extreme thermal conditions.

### Cell signaling

Cell signaling genes *ORMDL3*, *PAK4*, *TRIB3*, *SRY4*, *DUSP1*, *DUSP2*, *NCK2*, and *PTK6* that were upregulated at 25° (two-fold to 21-fold) largely function in the UPR, immunity or inflammation, the general stress response, and promotion or protection from apoptosis. ORMDL3 participates in calcium signaling in the endoplasmic reticulum and is associated with increased strength of the UPR (Cantero-Recasens *et al.* 2009). PAK4 protects the cell from apoptosis and reduces the effect of caspase 8 (Gnesutta *et al.* 2001; Gnesutta and Minden 2003). *TRIB3* is upregulated by stressful stimuli and, along with SRY4, regulates MAPK cell signaling (Kiss-Toth *et al.* 2004; Ord and Ord 2005; Cabrita and Christofori 2008). *DUSP1* and *DUSP2* encode serine/threonine kinases that are important for intracellular signaling and participate in inflammatory and immune signaling (Huang and Tan 2012). NCK2 is involved in intracellular signaling in both stress and normal conditions (Tu *et al.* 1998), as is PTK6. PTK6 is involved in DNA damage–induced apoptosis (Brauer and Tyner 2010). Much of the alteration in cell signaling genes relates directly to some aspect of the cellular stress response.

### Cell trafficking

The altered homeostatic and metabolic state of a highly stressed cell leads to a subsequent change in cellular trafficking of materials. One obvious change is an increased need for the transport of damaged proteins to the degradation machinery of the cell. Several genes related to cellular trafficking were upregulated in the 25° (two-fold to 15-fold) group of fish, including *HSP90B1*, *DNAJA1*, *SLC16A4*, *SLC16A2*, *AGL*, *XPO1*, *TRAM1*, and *RAB23*. *HSP90B1* encodes an endoplasmic reticulum protein that aids in the transport of proteins out of the ER, whereas upregulated *DNAJA1* is involved in the intracellular trafficking of vesicles that may be destined for the lysosome (Christianson *et al.* 2008; Parfitt *et al.* 2012). Upregulation of these two genes supports the assertion that the UPR is active in these juvenile Chinook salmon under thermal stress conditions and is further bolstered by the expression changes of other members of the same gene family (*e.g.*, HSP90A and DNAJ homologs) at lower treatment temperatures. There are several other trafficking proteins that are indirectly related to the stress response. Monocarboxylate transporters SLC16A4 and SLC16A2 transport small metabolic compounds within the cell and function in energy partitioning within the cell during the stress response (Halestrap 2013). Alteration in protein targeting to different organelles is also altered under stress conditions. AGL targets proteins to the nucleus, whereas XPO1 traffics proteins out of the nucleus in a signal-dependent manner (Moroianu *et al.* 1995; Ossareh-Nazari *et al.* 1997). Upregulated *SNTB1* is involved in transport into the mitochondria, whereas TRAM1 transports proteins across the ER membrane (Görlich *et al.* 1992; Nuttall *et al.* 1997). RAB23 is a small GTPase that participates in the trafficking within the cilia (Lim *et al.* 2011). The cellular processes that occur under thermal stress conditions differ greatly from normal cellular function and unsurprisingly lead to changes in cellular trafficking.

### Structure/cytoskeletal

There was a moderate difference in expression (two-fold to 10-fold) of genes involved in cytoskeletal structure and organization in the 25° group. *CCT4* and *CCT5* are genes required for the production of actin and tubulin, and TBCB is a cofactor that ensures proper folding of tubulin during production (Lopez-Fanarraga *et al.* 2001; Brackley and Grantham 2009). Several upregulated genes modulate the interactions between various cytoskeletal components but have no known association with the stress response. AIF1L cross-links actin filaments and is involved in actin bundling (Schulze *et al.* 2008). SYNPO organizes actin filaments in response to RHOA signaling (Asanuma *et al.* 2006). CNN2 (calponin 2) binds actin and is thought to aid in the regulation of cytoskeletal organization (Tang *et al.* 2006). *PDLIM1* encodes an adaptor protein that binds to cytoskeletal proteins (Sharma *et al.* 2011). These upregulated genes have not been previously associated with the stress response, but may play a role in stress-related structural changes in thermally stressed juvenile Chinook.

Certain upregulated cytoskeletal genes, such as *MMP13*, *TAGLN*, and *CDC42SE1*, have known associations with the cellular stress response. *MMP13* codes for the protein collagenase 3, which is involved in connective tissue degradation and cytoskeletal component turnover (Knauper *et al.* 1996). Several other MMP family members were notably downregulated (Table S1); therefore, the roles of other family members in this process are likely more complex than the upregulation of a single gene. *TAGLN* codes for a calponin family protein, transgelin, which gels actin filaments and is overexpressed in senescent cells (Thweatt *et al.* 1992; Assinder *et al.* 2009). CDC42SE1 mediates cytoskeletal scaffold changes in response to immune signaling through CDC42 (Pirone *et al.* 2000). The upregulation of these genes indicates that some degree of cellular restructuring may take place in response to thermal stress.

## Conclusion

### Conclusions and comparisons to existing studies

The results of this study corroborate previous findings on important heat stress–related genes and implicate many new genes in the thermal stress response of juvenile Chinook salmon. Some response pathways and individual genes characterize a common thermal stress response across fish species, life stage, and duration of thermal stress; however, many of the genes reported above are likely specific to the thermal stress response of Chinook salmon and the juvenile life stage. Chaperone-coding genes are universally upregulated in published studies of thermal stress and gene expression. Upregulation of members of the HSP90 gene group were found in the gill tissue of Chinook salmon in this study, as well as in other fish species exposed to either acute or prolonged periods at elevated temperatures (*e.g.*, goby in Buckley *et al.* 2006; killifish in Fangue *et al.* 2006; Arctic charr in Quinn *et al.* 2011; pink and sockeye salmon in Jeffries *et al.* 2014). Upregulation of *SERPINH1* has also been commonly found in other thermal stress studies of fish (*e.g.*, Logan and Somero 2010; Jeffries *et al.* 2012; Rebl *et al.* 2013). The UPR is strongly represented in the thermal stress literature as well. Logan and Somero (2010) found an upregulation in *PDIA4*, whereas we identified similar genes *PDIA6*, *PDIA4*-like, and *PDIA3*. Jeffries *et al.* (2014) found an increase in *FKBP10*, whereas we identified upregulation of related gene *FKBP4*.

Jeffries *et al.* (2014) study of gene expression in the gill tissue of chronically heat-stressed adult pink and sockeye salmon found the largest number of genes in common with the current study. In addition to chaperone genes in the HSP90 and HSP70 families, Jeffries *et al.* (2014) found inflammatory regulators involved with NF-κB activity and other inflammatory/immune regulatory genes. Jeffries *et al.* (2012) found that the increase in *ODC1* and *CEBPB* was the largest in moribund sockeye salmon, both of which were also upregulated in this study. They also identified several late-stage caspases involved in apoptosis, as did this study. Regardless of the difference in species, life stage, and duration of temperature exposure, many of the same functional pathways were upregulated in both the current and above studies, namely protein stabilization/degradation, apoptosis, and immunity/inflammation. In addition to these similarities, this study demonstrates an association of thermal stress in juvenile Chinook salmon with expression of some genes not previously linked to heat stress in salmonids, including cytoskeletal genes *CCT4*, *CCT5*, *TBCB*, *AIF1L*, *SYNPO*, *CNN2* and *PDLIM1*.

The protein p53 appears to play an important role in the thermal stress response observed in this experiment. Although p53 itself was not upregulated, many of the observed upregulated genes are regulated in some fashion by p53, including genes involved in apoptosis, cell proliferation, and oxidative stress.

These results bolster our understanding of the cellular processes that are important for coping with thermal stress, which is of increasing importance for the southern-most populations of Chinook salmon in the United States. As our climate changes and the demand for water increases due to human population growth, the remaining Chinook salmon in states such as California will almost certainly encounter warmer temperatures during their freshwater life stages.
